# Post-concussive complaints after mild traumatic brain injury associated with altered brain networks during working memory performance

**DOI:** 10.1007/s11682-015-9489-y

**Published:** 2015-12-14

**Authors:** Harm J. van der Horn, Edith J. Liemburg, Myrthe E. Scheenen, Myrthe E. de Koning, Jacoba M. Spikman, Joukje van der Naalt

**Affiliations:** 1 Department of Neurology, University of Groningen, University Medical Center Groningen, Hanzeplein 1, 9700 RB Groningen, The Netherlands; 2 BCN NeuroImaging Center and Department of Neuroscience, University of Groningen, University Medical Center Groningen, Groningen, The Netherlands; 3 Department of Neuropsychology, University of Groningen, University Medical Center Groningen, Groningen, The Netherlands

**Keywords:** Mild traumatic brain injury, Post-concussive complaints, Brain networks, Working memory

## Abstract

The aim was to investigate brain network function during working memory (WM) task performance in patients with uncomplicated mild traumatic brain injury (mTBI) in the sub-acute phase post-injury. We were particularly interested in differences between patients with (PCC-present) and without post-concussive complaints (PCC-absent). Fifty-two patients and twenty healthy controls (HCs) (matched for age, sex, education and handedness) were included. Two patient groups were created based on reported post-concussive complaints at two weeks post-injury: PCC-present (*n* = 32) and PCC-absent (*n* = 20). Functional MRI scans were made at approximately four weeks post-injury. Participants performed an *n*-back task consisting of three conditions (0-, 1- and 2-back) with increasing difficulty. General linear model analysis was performed to investigate activation patterns. Independent component analysis was used to identify brain networks. The frontal executive network (FEN), frontoparietal network (FPN) and default mode network (DMN) were selected for further analyses based on their highest task-relatedness. Task accuracy and reaction times were similar for patients with mTBI and HCs. During high WM load (2-vs.0-back contrast), mTBI patients exhibited lower activation within the medial prefrontal cortex compared to HCs. No differences were found between PCC-present and PCC-absent patients. Regarding network function, PCC-absent patients showed stronger deactivation of the DMN compared to PCC-present patients and HCs, especially during difficult task conditions. Furthermore, functional connectivity between the DMN and FEN was lower in PCC-absent patients compared to PCC-present patients. Interestingly, network function did not differ between PCC-present patients and HCs, suggesting that non-injury related factors may underlie post-concussive complaints after mTBI.

**Electronic supplementary material:**

The online version of this article (doi:10.1007/s11682-015-9489-y) contains supplementary material, which is available to authorized users.

## Introduction

Annually, millions of people sustain a traumatic brain injury (TBI), with the vast majority (85–90 %) incurring a mild injury (mTBI) (Corrigan et al. 2010). Patients with mTBI frequently report cognitive and/or affective complaints, which most often resolve within weeks, but may persist for months to years in a subgroup of patients (Willer & Leddy, 2006). However, with conventional magnetic resonance imaging (MRI) sequences, usually no lesions are detected that might explain these complaints in patients with mTBI (Bazarian et al. 2006; Iverson et al. 2000).

Functional MRI (fMRI) studies using working memory (WM) paradigms have provided more insight into the concept of mTBI. WM involves short-term storage and manipulation of information and is considered crucial for higher-order cognitive functioning (Owen et al. 2005). Already in 1999, it was demonstrated that altered brain activation patterns may be related to cognitive complaints in patients with mTBI, despite the fact that WM performance was unimpaired (McAllister et al. 1999). Since then, several studies have been published on this subject, with varying results (Mayer et al. 2015a). Some studies have reported higher activation, whereas others reported lower activation post-mTBI, and differences may be partly explained by task design and difficulty (Bryer et al. 2013). However, to date, there is still no clear explanation for the occurrence of post-concussive complaints after mTBI.

In recent years, evidence has accumulated that dysfunction of brain networks plays a major role in the pathophysiology of mTBI. Studies have reported alterations within the default mode network (DMN) and stronger connectivity between the DMN and (parts of) executive networks during resting conditions (Borich et al. 2015; Mayer et al. 2011; Sours et al. 2013; Zhou et al. 2012; Zhu et al. 2015). However, in contrast to severe TBI, no study so far has investigated brain network function during WM performance in patients with mTBI (Palacios et al. 2012). Furthermore, the link between network function and the presence or absence of post-concussive complaints remains unclear.

In the present study, we investigated the relationship between network function, WM performance and post-concussive complaints in the sub-acute phase after mTBI.

## Methods

### Participants

This study was conducted as part of a larger prospective multicenter follow-up study (UPFRONT study). Fifty-five patients (age 18–65 years old) with mTBI were prospectively included at the University Medical Center Groningen, The Netherlands (a level 1 trauma center) between March 2013 and February 2015. The diagnosis of mTBI was based on a Glasgow Coma Score of 13–15 and/or loss of consciousness ≤30 min (Vos et al. 2012). The following exclusion criteria were applied: lesions on admission computed tomography (CT) scans, neurological or psychiatric comorbidity, prior admission for TBI, drug or alcohol abuse, mental retardation and contraindications for MRI (implanted ferromagnetic devices or objects, pregnancy or claustrophobia). A group of twenty healthy controls (HCs) was recruited among social contacts and via advertisements. Healthy controls did not have any history of TBI or other neurological or psychiatric diseases, and did not suffer from current psychiatric or neurological conditions. MTBI patients and HCs were group-matched for age, gender, educational level and handedness.

### Post-concussive complaints

Two subgroups, patients with (PCC-present) and without (PCC-absent) post concussive complaints, were created based on their answers on a post-concussive questionnaire administered at two weeks post-injury. This questionnaire is derived from the Rivermead Post-concussion symptoms Questionnaire (RPQ) (King et al. 1995) and composed of 19 complaints. Pre-injury as well as current complaints were measured on a scale from 0 to 2 (0 = never, 1 = sometimes, 2 = often). PCC-present was defined as ≥3 complaints, with at least one complaint within the cognitive or affective domain. PCC-absent was defined as <3 complaints. The presence of complaints following the moment of scanning was determined based on answers on follow-up questionnaires administered at three to six months post-injury and data from outpatient appointments.

In addition, feelings of anxiety and depression post-mTBI were assessed using the Hospital Anxiety and Depression Scale (HADS) (Zigmond & Snaith, 1983). Group analyses were performed on raw anxiety (HADS-A) and depression (HADS-D) scores. A cut-off score of ≥8 was used as an indicator of anxiety or depressive disorder (Bjelland et al. 2002).

### MRI acquisition

Images were acquired at approximately four weeks post injury by using a 3.0 T Philips Intera Achieva MRI scanner (Phillips Medical Systems, Best, The Netherlands). A high resolution transversal T1-weighted sequence image was made for anatomical reference with the following parameters: repetition time (TR) 9 ms; echo time (TE) 3.5 ms; flip angle (FA) 8°; field of view (FOV) 256 × 232 mm; voxel size 1x1x1 mm). Functional MRI was acquired using gradient echo planar imaging with the following parameters: TR 2000 ms; TE 20 ms; FOV 224x224mm; voxel size 3.5 × 3.62 × 3.5 mm. To account for T1 equilibrium effects, image acquisition was preceded by a preparation phase. The following sequences were performed to examine post-traumatic lesions: a coronal T2-gradient echo (TR 875 ms; TE16ms; FOV 230 × 183.28 mm; voxel size 0.40 × 1.12x4mm) and a transversal susceptibility weighted imaging (TR 35 ms; TE 10 ms; FOV 230 × 183.28 mm; voxel size 0.90 × 0.90x2mm). Microbleeds (≥2; 1-10 mm) were observed in 40 % of patients, with no differences between PCC-present and PCC-absent patients.

### FMRI paradigm

A verbal *n*-back with visual stimuli was presented by using E-prime v2 (Psychology Software Tools, Sharpsburg, PA, USA) on a screen visible via a mirror on top of the head coil. Three conditions (0-, 1- and 2-back) had to be performed, in which a sequence of letters was presented (stimulus time 500 milliseconds (ms), inter-stimulus interval 1000 ms). During the 0-back condition, patients had to respond by button presses when one specific letter (“X”) appeared, and during the 1- and 2-back conditions if the presented letter matched the letter respectively one or two steps back in the sequence. The task consisted of 12 pseudo-randomized blocks (4 blocks of 36 s per condition), with a fixation period between conditions. Prior to scanning, task instruction was given and this instruction was repeated in the MRI-scanner before the onset of the task. Task accuracy was defined as the percentage of correct responses for every condition. Three patients were excluded from final analyses due to difficulties with understanding the task and/or aberrant task performance (i.e. <50 % correct responses on the 0- or 1-back condition, excessive number of false presses). Task accuracy could not be calculated for seven of the remaining patients (four PCC-present, three PCC-absent) and six HCs, due to initial technical problems with logging procedures. However, we included these participants in the final fMRI-analyses, because they appeared to perform adequately based on the available data and the interview after the experiment. To account for possible inconsistencies, we also conducted the analyses with exclusion of these participants.

### FMRI preprocessing

Preprocessing was performed using Statistical Parametric Mapping (SPM) v12 (Wellcome Trust Centre for Neuroimaging, University College London, London, England) implemented in Matlab v2011b (MathWorks, Natick, MA, USA). Functional images were realigned, co-registered with the individual participant’s T1-weighted image, normalized using diffeomorphic nonlinear registration tool (DARTEL) (isotropic voxels of 2x2x2mm) and smoothed (8 mm full-width at half maximum (FWHM) Gaussian kernel).

### General linear model

For first level general linear model (GLM) analyses, task conditions were modeled as a boxcar function convolved with the hemodynamic response function in SPM12, with inclusion of six motion parameters, their derivatives, and a 176 s high pass filter. The following t-contrasts were made for every participant: 1- vs. 0-back (low WM load), 2- vs. 0-back (high WM load) and 2- vs. 1-back (moderate WM load). Subsequently, these contrasts were analyzed on second level in SPM.

### Network analyses

To identify functional brain networks, independent component analysis (ICA) was performed using the Group ICA of fMRI Toolbox (GIFT; version 3.0a, MIALAB Software) implemented in Matlab (Calhoun et al. 2001). The number of independent components (ICs) was estimated using Maximum Description Length (MDL) and Akaike’s criteria (Li et al. 2006). Principal component analysis (PCA) was run for data reduction purposes (step one: 60 principal components, step two: 40 components). Group ICA was performed using the Infomax algorithm, with the ICASSO approach to realize IC stability (20×) (Himberg et al. 2004). Spatial-temporal regression was used for back-reconstruction. Forty ICs were extracted. Components reflecting artifacts, including head motion, physiological and scanner noise, cerebrospinal fluid and white matter were discarded after inspection of spatial maps and power spectra (Allen et al. 2011). Identification of artifact components was done independently by H.J.v.d.H. and E.J.L. and subsequently discussed until consensus was reached. The decision point of removal was based on spatial overlap with gray matter, spatial outline, and dominance of low-frequencies in the power spectrum, and decided by expert opinion.

To calculate the relationship between task conditions and IC time-courses, first level SPM models were entered in the temporal sorting (i.e. regression) tool in GIFT. For every subject, three beta values per IC were obtained, reflecting the degree to which an IC was modulated by the three different task conditions. Positive modulation (roughly) corresponds with activation and negative modulation corresponds with deactivation. The ICs were sorted from highest to lowest task-relatedness according to the *R* ^2^-values from temporal regression in the total dataset. We selected the three networks that showed the highest task-relatedness for further analyses. Although this method has been used in earlier research, it is relatively new in the field of mTBI (Kullmann et al. 2013; Palacios et al. 2012).

Within-network functional connectivity (FC) of ICs was examined on second level in SPM12, which yields information about the voxel-wise contribution to the spatial map a certain component. To determine between-network FC, time-courses were analyzed using the Functional Network Connectivity (FNC) toolbox v2.3 implemented in Matlab (Jafri et al. 2008). To our knowledge, this is a relatively new method to investigate between-network FC in patients with mTBI. A Butterworth band-pass filter (0.013–0.24 Hz) and a lag-shift of three seconds were applied. Correlations were calculated between entire time-courses and not for each task condition separately. Absolute correlation values were used to take into account negative correlations between networks, and these values were Fisher z-transformed and imported into SPSS for statistical analyses.

### Statistics

Participant characteristics were analyzed with the Statistical Package for Social Sciences (SPSS) v22.0 (IBM Corp, Armonk, NY, USA).

Group differences in first level contrasts were analyzed in SPM12 using flexible factorial designs with the following factors: *subject* (to control for between-subject variation), *group* (HC, PCC+ and PCC-) and *load* (1-vs. 0-, 2-vs. 0- and 2-vs. 1-back contrasts). A priori t-contrasts were made to compare the total mTBI- and HC-group. Regarding subgroups, F-tests were conducted and if there was a significant group effect post-hoc t-contrasts were made. The threshold for group differences was set at *p*_uncorrected_ < 0.001 with family wise error (FWE) - cluster correction (*p* < 0.05) for multiple comparisons based on random field theory. For the networks identified with ICA, beta-weights from temporal sorting were entered in a repeated measures permutation test in MATLAB with 5000 random permutations. The α-level of 0.05 for group differences was adjusted for multiple testing (3 networks × 3 conditions =9 tests) using the false discovery rate (FDR) procedure (corrected α-level = 0.05 x rank *p*-value/9) (Benjamini & Hochberg, 1995). Differences in within- and between-network FC between the total mTBI and HC group were examined using (a priori) independent sample t-tests. Regarding subgroups, one-way ANOVA was conducted, followed by two-sample t-tests in case of significant group effects. Since multiple components were assessed for group differences in within-network FC, the statistical threshold was set at *p*_uncorrected_ < 0.001, cluster corrected *q*FDR < 0.01, k > 10 voxels, based on a study by Veer et al*.* (Veer et al. 2010). For between-network FC, a threshold of *p* < 0.05 was used with FDR correction for six tested component pairs.

Since there was a difference in the percentage of male and female patients between subgroups, ANCOVA and repeated measures ANCOVA (*p* < 0.05) were performed in SPSS to examine the influence of sex on fMRI differences between subgroups.

## Results

### Participant characteristics

Participant characteristics are listed in Table 1. Although the total group of mTBI patients was matched with HCs, the group of PCC-absent patients contained more male subjects compared to the PCC-present group (χ^2^ = 7.606, *p* = 0.006). For PCC-present patients, the average number and severity of complaints was 9.6 and 12.8, respectively. The five most frequently reported complaints at two weeks were fatigue (reported by 91 %), headache (84 %), noise intolerance (84 %), dizziness (81 %) and forgetfulness/slowness/drowsiness (all 77 %). Ninety-seven percent (*n* = 31) of the PCC-present group still reported ≥3 complaints and three percent (*n* = 1) reported one complaint at follow-up after the scan.

**Table 1:** Participant characteristics

	PCC-present (*n* = 32)	PCC-absent (*n* = 20)	HC (*n* = 20)	*p*-value
Age, y, mean (range)	37 (19–63)	39 (20–64)	34 (18–61)	0.820^a^
Sex, % male	53	90	65	0.023^b^
Education level, median (range)^c^	6 (4–7)	6 (2–7)	6 (5–7)	0.277^b^
Handedness, % right	91	80	85	0.551^b^
MRI: days from injury, d, median (range)	32 (22–62)	33 (22–69)	N/A	0.528^d^
GCS-score, median (range)	14 (13–15)	15 (13–15)	N/A	0.063^b^
Injury mechanism:				
Traffic (%)	53	50	N/A	0.826^b^
Falls (%)	38	45	N/A	0.504^b^
Sports (%)	3	0	N/A	0.405^b^
Assault (%)	3	0	N/A	0.405^b^
Other (%)	3	5	N/A	0.763^b^
HADS-scores				
HADS-A, mean (SD)	5.3 (4.0)^e^	2.5 (2.5)	N/A	0.007^d^
HADS-D, mean (SD)	5.5 (4.1)^e^	1.0 (1.7)	N/A	<0.001^d^
*n*-back task accuracy				
% Correct 0-back, mean (SD)	97.4 (3.2)	94.5 (9.6)	98.2 (3.1)	0.514^a^
% Correct 1-back, mean (SD)	94.9 (6.3)	95.4 (5.2)	95.4 (5.8)	0.984^a^
% Correct 2-back, mean (SD)	83.0 (10.8)	75.2 (15.2)	82.1 (10.1)	0.111^a^
*n*-back task reaction time:				
RT 0-back, ms, mean (SD)	418.8 (38.8)	423.3 (55.9)	429.5 (47.0)	0.778^f^
RT 1-back, ms, mean (SD)	496.7 (65.0)	481.4 (103.1)	484.4 (68.8)	0.789^f^
RT 2-back, ms, mean (SD)	587.9 (90.9)	612.5 (138.6)	600.0 (84.7)	0.393^f^

Abbreviations: *MRI* Magnetic Resonance Imaging; *GCS* Glasgow Coma Scale; *N/A* not applicable; *HADS* Hospital Anxiety and Depression Scale.; *RT* reaction time

^a^Kruskal-Wallis test

^b^Pearson’s chi-square test

^c^Education level was based on a Dutch classification system, according to Verhage (1964), ranging from 1 to 7 (highest)

^d^Mann-Whitney *U* test

^e^Data regarding HADS were collected for 91 % of the PCC-present patients

^f^One-way ANOVA

PCC-present patients scored higher on the HADS-A (*p* = 0.007) and HADS-D (*p* < 0.001) items than PCC-absent patients. Within the PCC-present group, 28 % of the patients scored above the cut-off for affective disorder with anxiety (*n* = 2), depression (*n* = 4) or both (*n* = 3). None within the PCC-absent group scored above the cut-off for anxiety or depression.

### General linear model

**Fig. 1:**
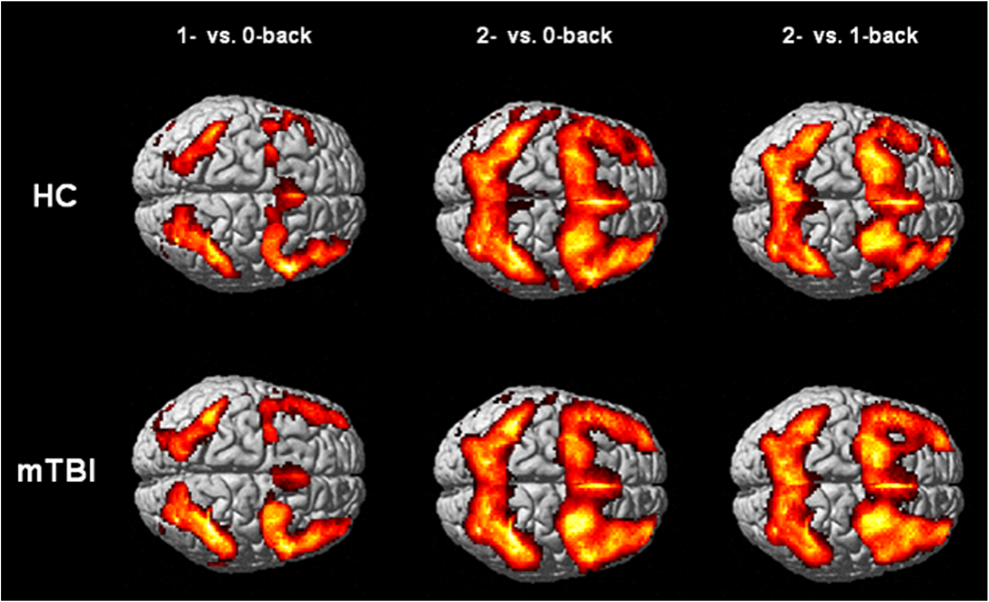
Working memory activation patterns for healthy controls (HC) and patients with mTBI (*p*_uncorrected_ < 0.001, cluster-level FWE-corrected *p* < 0.05)

**Fig. 2:**
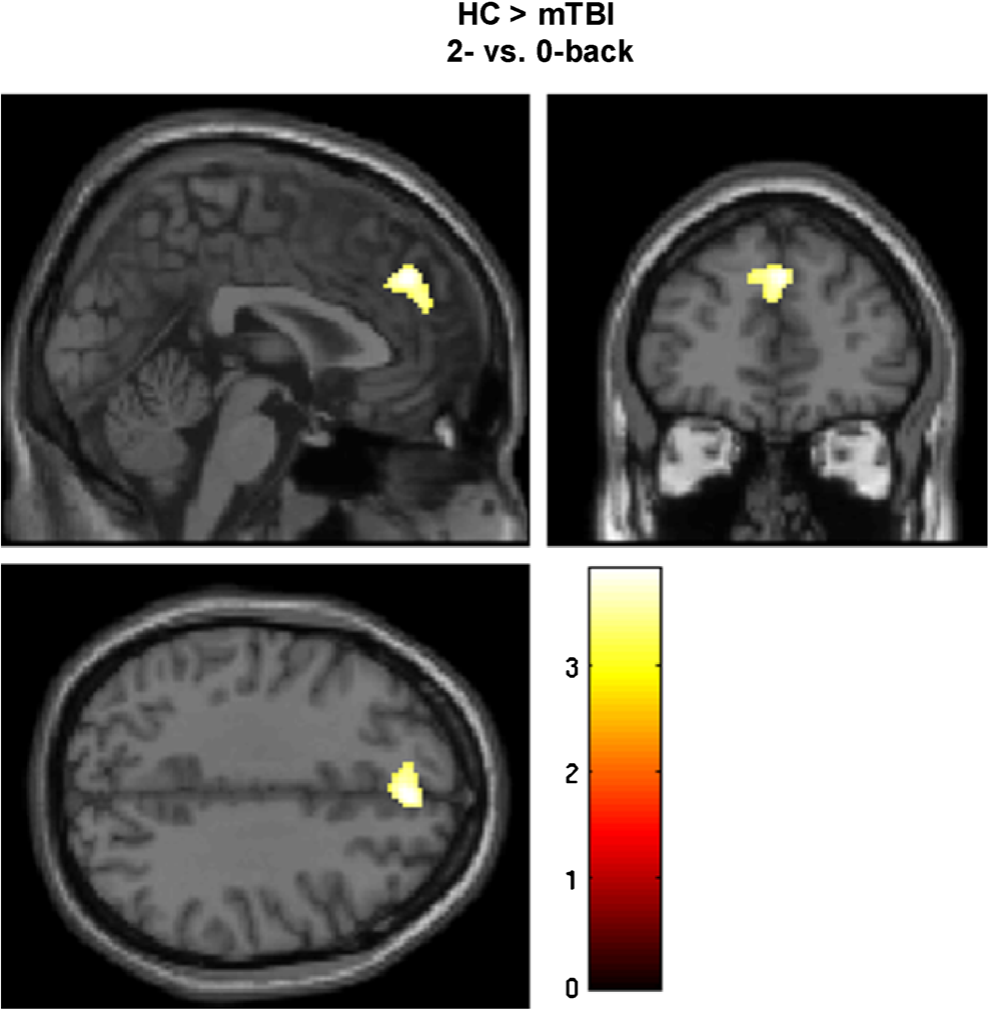
Differences in working memory activation between HC and patients with mTBI during high working memory load. In mTBI patients, a significant cluster of lower activation was found within the medial prefrontal cortex (peak MNI-coordinates: 2, 44, 36) compared to HC (*p*_uncorrected_ < 0.001, cluster-level FWE-corrected *p* = 0.045)

Activation patterns for HCs and patients with mTBI are depicted in Fig. 1. With respect to the 2- vs. 0-back contrast, significantly lower activation was found in the medial prefrontal cortex in patients with mTBI compared to HCs (Fig. 2). After exclusion of participants without available detailed task accuracy data, a trend towards lower activation of a cluster within the medial prefrontal cortex was found in patients with mTBI (cluster-level FWE-corrected *p* = 0.067). Instead, significant clusters of lower activation were observed within the cerebellar vermis (peak MNI-coordinates: 0, −54, −6, cluster-level FWE-corrected *p* = 0.038) and left supramarginal gyrus (peak MNI-coordinates: -50, −40, 48, cluster-level FWE-corrected *p* = 0.016). The F-test did not show a significant effect of subgroup regarding the 2- vs. 0-back contrast.

For the 1- vs. 0-back and 2- vs. 1-back contrasts, no differences were found between mTBI patients and HCs. Furthermore, F-tests did not reveal significant effects of subgroup. These results remained non-significant after exclusion of patients without detailed task accuracy data.

### Network identification

**Fig. 3:**
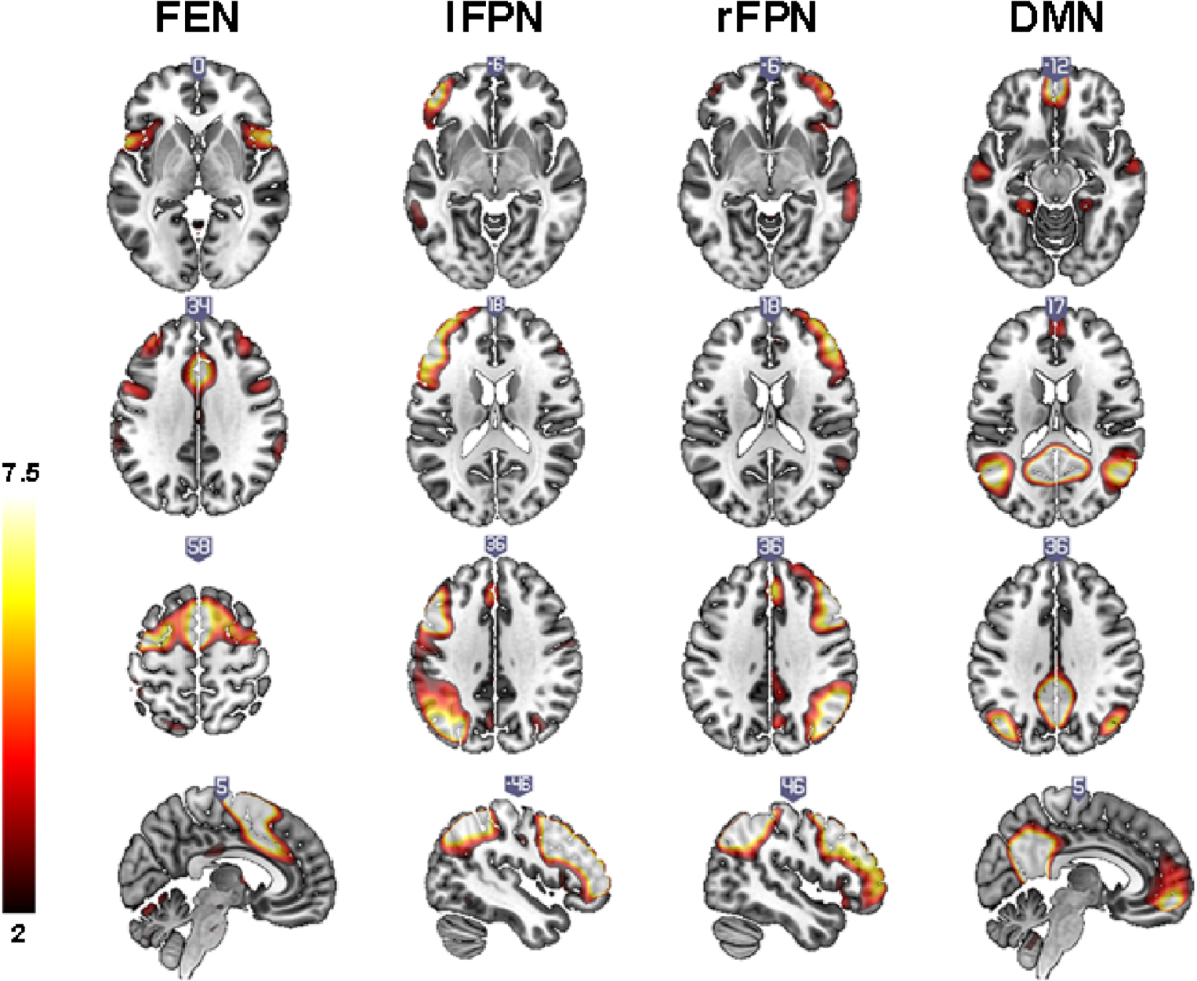
Spatial maps of the frontal executive network (FEN), frontoparietal network (FPN) and default mode network (DMN). Axial slices are displayed according to neurological convention

**Fig. 4:**
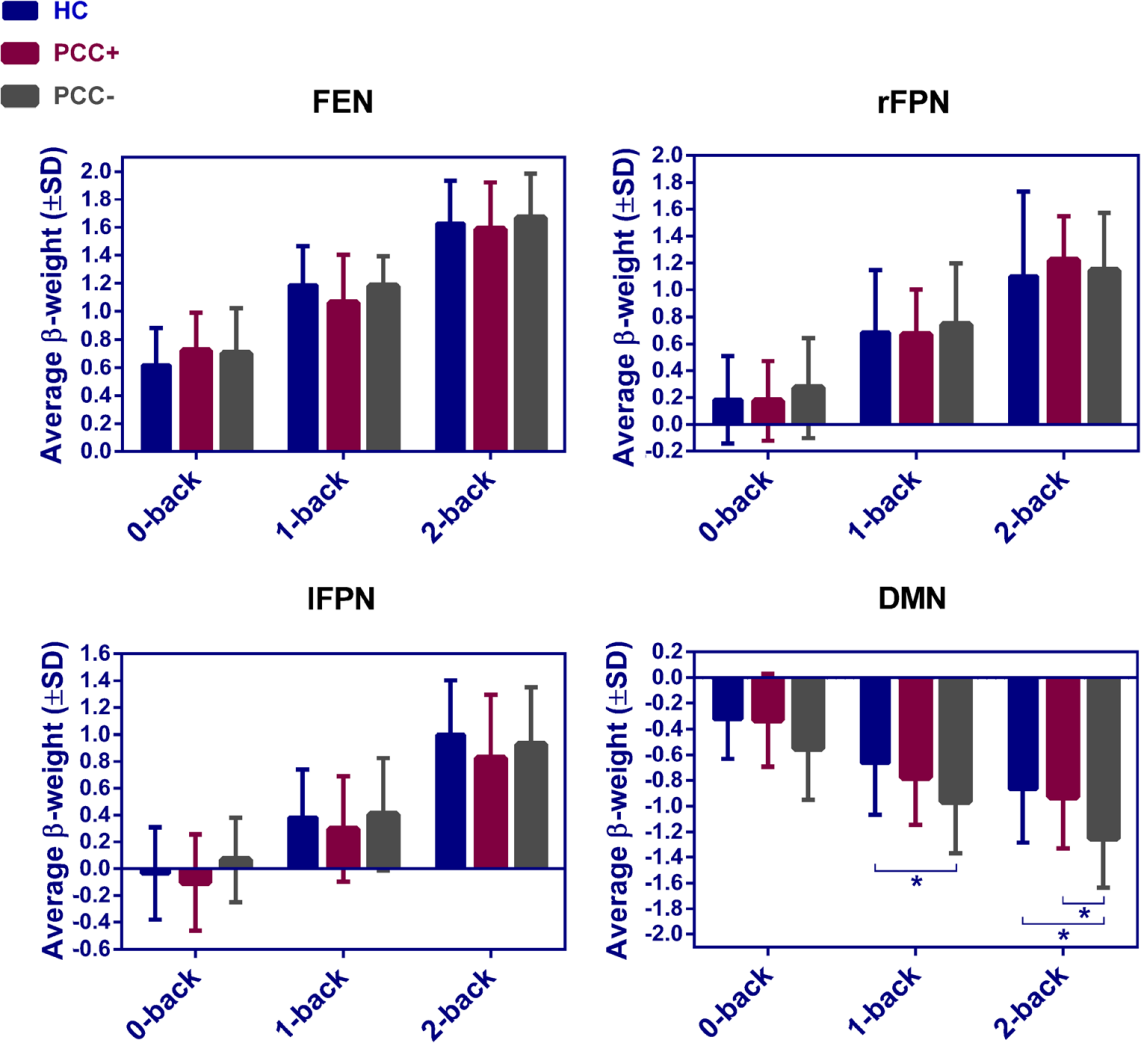
Average beta-weights expressing network activation or deactivation during working memory performance for HC, PCC-present (PCC+) and PCC-absent (PCC-) patients. Asterisks indicate significant group differences (*p* < 0.05 after FDR correction)

Forty ICs were extracted. Nineteen ICs reflected artifacts and were discarded. From the remaining functional networks, three were selected for further analyses based on their highest task-relatedness, which we will refer to as the frontal executive network (FEN), frontoparietal network (FPN; consisting of a left and right lateralized component) and DMN (Fig. 3 and Suppl. Table 1). The FEN and FPN were activated (increasing beta-weights) and the DMN was deactivated (decreasing beta-weights) with increased task difficulty (Fig. 4).

### Group comparisons of network activation or deactivation

Comparison of beta-weights from temporal sorting showed no significant differences in network activation or deactivation between HCs and the total group of mTBI patients. During the 2-back condition, PCC-absent patients showed stronger deactivation of the DMN compared to PCC-present patients (*p* = 0.0024; Fig. 4). A similar trend was observed for the 0- and 1-back conditions (*p* < 0.05, non-significant after FDR correction). Stronger deactivation was also found during the 1- and 2- back conditions in PCC-absent patients compared to HCs (*p* = 0.0098 and *p* = 0.002, respectively), with a similar trend for the 0-back condition (*p* < 0.05, non-significant after FDR correction). Results remained consistent after exclusion of participants without available detailed task accuracy data. Since the PCC-absent group contained a relatively high percentage of male patients, a repeated measures ANCOVA (within-subject factor: *condition*; between-subject factor: *subgroup*) with sex as a covariate was performed, which confirmed the significant effect of subgroup on DMN deactivation during working memory performance (*p* = 0.029). For the FEN and FPN, no significant differences in activation were present between groups. These findings remained non-significant after exclusion of participants without detailed task accuracy data.

### Group comparisons of functional network connectivity

**Fig. 5:**
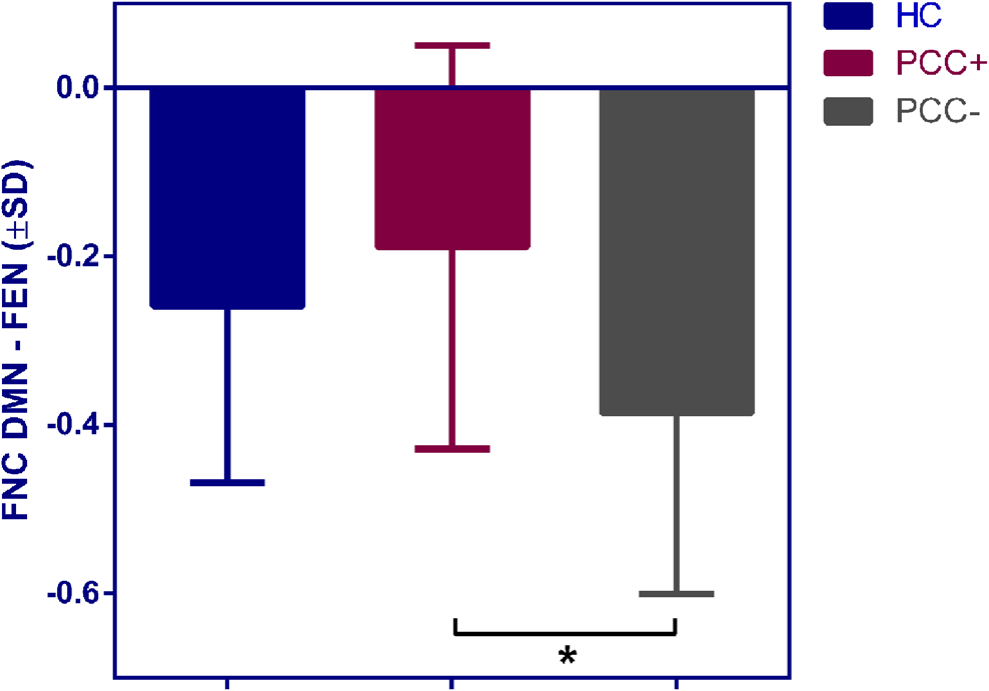
Functional network connectivity (FNC) of the DMN – FEN pair for HC, PCC-present (PCC+) and PCC-absent (PCC-) patients. Asterisk indicates a significant group difference (*p* < 0.05 after FDR correction)

Functional connectivity between the DMN and FEN was significantly lower in PCC-absent patients compared to PCC-present patients (*p* = 0.004; Fig. 5). An additional ANCOVA with sex as a covariate showed a similar effect of subgroup on functional connectivity between these networks (*p* = 0.035). No further group differences were found regarding within- and between-network functional connectivity. Results remained consistent after exclusion of participants without available data regarding task accuracy.

## Discussion

This is the first study that assessed the relationship between brain network function, WM performance and post-concussive complaints in the sub-acute phase after mTBI. In the total group of patients with mTBI, lower activation was found within the medial prefrontal cortex during high WM load, despite normal task accuracy and reaction times. Regarding subgroups, ICA revealed that DMN function and the interaction between the DMN and FEN were different between patients with and without post-concussive complaints. Altogether, these results might provide new insights into the concept of mTBI and post-concussive complaints.

So far, several studies have investigated WM performance in mTBI, with varying results (Bryer et al. 2013; Mayer et al. 2015a). In general, stronger activation was observed during high task difficulty compared to healthy controls, which could indicate increased mental effort to maintain normal task accuracy, leading to mental fatigue (McAllister et al. 1999; McAllister et al. 2001; Smits et al. 2009). Moreover, with increasing task difficulty patients with mTBI were not able to sufficiently recruit brain areas for WM performance, which was reflected by lower activation compared to controls (McAllister et al. 2001). However, this finding mostly pertains to lateral prefrontal and parietal areas and the supplementary motor area. In the present study, we demonstrated lower activation within the medial prefrontal cortex during high WM load in mTBI patients compared to healthy controls. This region is important with respect to executive functioning, but also with regard to emotion regulation (Euston et al. 2012; Messina et al. 2015). Furthermore, the medial prefrontal cortex is a core area of the DMN and an important relay station in the interaction between the DMN and other (executive) brain networks (Buckner et al. 2008; Seeley et al. 2007). Our findings may thus reflect stronger deactivation of the DMN during working memory performance. Since the prefrontal cortex is often affected by TBI, it can be hypothesized that changes in function of the medial prefrontal cortex are related to post-concussive complaints and emotion regulation deficits after mTBI (van der Horn et al. 2015). In particular, dysfunction of this area may lead to impaired dynamics between the DMN and executive networks, resulting in cognitive and affective complaints. Our network analyses revealed significant results that are in line with this hypothesis.

Strikingly, network analyses showed that PCC-absent patients exhibited stronger deactivation of the DMN during WM performance compared to PCC-present patients. To our knowledge no study so far used ICA to investigate brain networks during WM performance in patients with mTBI. A recent report has shown stronger deactivation in a particular area of the DMN (i.e. posterior cingulate cortex) during an *n*-back task in patients without cognitive complaints compared to patients with complaints at one week post-injury (Wylie et al. 2015). One study used ICA to study WM performance in patients with more severe TBI in the chronic phase after injury (Palacios et al. 2012). In these patients, higher DMN activity is associated with lapses in attention and impaired cognitive functioning (Bonnelle et al. 2011; Palacios et al. 2012). This phenomenon is also known as default mode interference, which is supposed to be related to ineffective switching between the DMN and networks involved in executive functioning (Menon & Uddin, 2010; Sonuga-Barke & Castellanos, 2007). In order to adequately perform a cognitive task, not only executive networks need to be activated, but it is imperative that the DMN is adequately deactivated to prevent default mode interference and to facilitate network switching (Koshino et al. 2014; Sonuga-Barke & Castellanos, 2007). A third network, the salience network, modulates the interaction between the DMN and executive networks during cognitive tasks and thereby exerts a supporting role in network switching (Seeley et al. 2007; Sridharan et al. 2008). Our current findings suggest that PCC-absent patients are better at suppressing the DMN and switching to an executive state, resulting in fewer complaints. On the other hand, it might be possible that the presence of complaints in PCC-present patients impedes DMN deactivation. We also observed stronger coupling of the DMN and FEN in PCC-present patients compared to PCC-absent patients, suggestive of problems with network switching. This is consistent with resting state fMRI studies that showed that stronger connectivity between the DMN, executive networks and salience network is related to cognitive complaints (Mayer et al. 2011; Sours et al. 2013). Interestingly, the FEN we identified during WM performance also contains the insulae and anterior cingulate cortex, which are areas that are often described as parts of the salience network (Seeley et al. 2007). This finding further indicates that network switching may be involved in post-concussive complaints. It is plausible that PCC-present patients need stronger top-down control of the DMN by executive networks in collaboration with the salience network in order to suppress internal thoughts and to switch to an externally directed mental state that is necessary for WM performance (Sours et al. 2013). For future research, it may be worthwhile to use a combination of cognitive paradigms and resting state fMRI to shed more light on the relationship between network switching and post-concussive complaints.

The question is whether the observed findings regarding network function are related to structural abnormalities. Recent diffusion tensor imaging (DTI) studies have not shown clear evidence that patients with and without complaints differ with respect to micro-structural injury (Lange et al. 2015; Waljas et al. 2015). A recent systematic review has also indicated that common *subjective* symptoms after mTBI, which we therefore refer to as complaints, are not necessarily caused by brain injury per se, but also occur in the general population and after non-head injuries (Cassidy et al. 2014). These reports suggest that differences in network function between patients with and without complaints may be strongly associated with non-injury related factors, such as pre-injury mental problems, personality factors and current life stress. We deem it plausible that network findings after mTBI reflect inter-individual (perhaps pre-injury) differences in for example emotion regulation abilities and coping, which determine the level of mental distress and consequently the expression of complaints (van der Horn et al. 2015). We have found that levels of anxiety and depression were significantly higher in PCC-present patients than in PCC-absent patients, which is consistent with the idea that emotion regulation abilities play an important role in the development of post-concussive complaints (van der Horn et al. 2015). Chen et al. demonstrated that depressive complaints after mTBI are associated with higher activity in DMN related areas during WM performance (Chen et al. 2008). It can be hypothesized that higher DMN activity in patients with mTBI is associated with rumination, similar to what happens in patients with a major depressive disorder (Whitfield-Gabrieli & Ford, 2012). Additionally, in healthy individuals higher activity of the DMN is related to worrying and higher neuroticism scores, and predisposes to anxiety and depression (Servaas et al. 2014). Although speculative, we propose that stronger suppression of the DMN during WM performance in PCC-absent patients may in turn be related to more effective emotion regulation, which also relies on adequate control of the DMN via executive networks (Cole et al. 2014). PCC-absent patients may require less top-down control of the FEN to suppress the DMN, reflected by lower connectivity strength between these networks compared to PCC-present patients.

Surprisingly, our findings indicate that PCC-present patients may be more comparable to healthy controls than to PCC-absent patients. Most patients with mTBI are known to develop some post-concussive complaints (McMahon et al. 2014; Waljas et al. 2015). Up to 12 months after injury in 80 % of patients at least one complaint is present, with a mean of six complaints overall (Track-TBI) (McMahon et al. 2014). It is important to realize that these complaints are frequently reported in the absence of objective functional deficits and are likely reflecting subjective feelings of decreased wellbeing. The fact that healthy controls may report similar complaints underlines the possibility that non-injury related factors, such as personality style and emotion regulation abilities, are predominantly involved the development of complaints after mTBI (Cassidy et al. 2014; Waljas et al. 2015). So far it is not clear which patients will develop complaints and who will be able to resume activities despite complaints. Most studies focus on predictive factors for the development of complaints and contain a relatively high percentage of patients with complaints reflecting the overall distribution of complaints in mTBI. However, it might be even more interesting/informative to investigate those patients who do not develop complaints at all, which has not been done until now. Perhaps these patients have specific personality characteristics that facilitate good recovery. The relative overrepresentation in our cohort of such patients without complaints might be the reason that for the first time this difference in subpopulations of mTBI is highlighted. Whether or not our findings are indicative of compensatory mechanisms or pre-injury network characteristics related to specific personality traits has to be further investigated.

Furthermore, few differences were observed when directly comparing fMRI results between the total group of mTBI patients and the healthy control group. We included patients with a significant number of complaints and a group of patients with no complaints at all, reflecting either end of the spectrum of complaints, which might account for the negative findings regarding the total mTBI group. In fact, this further underlines the possibility that the injury itself may be less influential in the development of post-concussive complaints than pre-morbid personality characteristics and emotion regulation abilities. In this respect, it could be argued that differentiation of patients with mTBI based on anxiety and/or depression instead of post-concussive complaints might be more informative. Certain other methodological aspects may have played a role in our negative findings. First, we applied a relatively stringent statistical threshold compared to previous studies. Creating the optimal balance between type-1 and type-2 errors is (still) a subject of debate in neuroimaging (Bennett et al. 2009; Lieberman & Cunningham, 2009). Since we included fairly large patient samples, appropriate correction for multiple comparisons was required. To avoid missing possible (clinically) relevant findings, we reported results without corrections at voxel-level and only applied family wise error correction at cluster-level. Absence of network findings may have been influenced by the type of network analysis technique that we used. Both ICA (Mayer et al. 2015a, b; Shumskaya et al. 2012; Zhou et al. 2012) and seed-based methods (Johnson et al. 2012; Mayer et al. 2011; Sours et al. 2013; Sours et al. 2014; Zhu et al. 2015) are frequently used to investigate network function after mTBI. We chose to perform ICA, because it may be more suitable and informative considering the heterogeneous nature of mTBI; however, we realize that this method has its disadvantages compared to seed-based methods (Cole et al. 2010). Second, task conditions used in our study were relatively difficult, considering the high stimulus presenting rate compared to other studies (1/1500 ms vs. 1/3000 ms) (Chen et al. 2012; McAllister et al. 1999; McAllister et al. 2001; Smits et al. 2009). This may also be the reason that we found lower instead of higher activation during the 2- vs. 0-back contrast in mTBI patients, similar to that observed during a 3- vs. 2-back contrast in a study by McAllister and colleagues (McAllister et al. 2001). However, one has to take into account the possibility of over-subtraction in causing their findings, considering that hyper-activation was already observed during the 2- vs. 1-back contrast.

The present study, inevitably, holds limitations. First, we did not administer HADS questionnaires in the healthy control group, although during the interview before inclusion participants were screened for psychiatric problems, including depression, which were not reported. Interestingly, studies have shown HADS-A and HADS-D scores in healthy subjects that are comparable to those reported by patients in our PCC-present group (Bocerean & Dupret, 2014; Michopoulos et al. 2008; Spinhoven et al. 1997). We also acknowledge that the absence of an early neuropsychological assessment prevents us from making a clear statement about the cognitive abilities of this study population. However, task-performance accuracy and reaction times during the *n*-back task did not differ between mTBI patients and healthy controls, and most other studies did not show deficiencies on neuropsychological tests in patients with mTBI (Chen et al. 2012; McAllister et al. 1999), or showed only subtle deficiencies reflected in slightly higher reaction times in the sub-acute and chronic stage (Chen et al. 2007; McAllister et al. 2001). As post-concussion questionnaires were administered at two weeks post-injury, in theory patients with complaints at two weeks may have been (partially) recovered at time of scanning at four weeks post-injury. However, because of the high complaint levels at two weeks (average 9.6), the relatively short interval between questionnaires and scanning (1–2 weeks), and the fact that everyone in this group still reported complaints at follow-up after scanning, we have strong reasons to assume that patients were still suffering from significant complaints at the time of scanning.

To conclude, the present study demonstrates lower medial prefrontal activity during difficult WM conditions in the sub-acute phase after mTBI. Furthermore, this is the first study that used ICA to show network changes during WM task performance in patients with mTBI. Absence of post-concussive complaints was associated with specific patterns of network activity and connectivity, especially with regard to the DMN and FEN, and network measures in PCC-present patients were comparable to those in healthy controls. Whether these findings reflect compensatory mechanisms and/or non-injury related factors has to be further investigated. Longitudinal designed neuroimaging studies, that also examine emotion regulation and include DTI to assess structural network integrity, may provide more clarity on the nature of network alterations after mTBI.

## Electronic supplementary material

Suppl. Table 1 (DOCX 27 kb)
