# Supplementary material for: Post-concussive complaints after mild traumatic brain injury associated with altered brain networks during working memory performance
Source: Brain Imaging Behav. 2015 Dec 14;10(4):1243–53. doi: 10.1007/s11682-015-9489-y (PMC5167217; doi:10.1007/s11682-015-9489-y)
Supplement: Supplementary file 1 — (DOCX 27 kb) [file 11682_2015_9489_MOESM1_ESM.docx]

**Suppl. Table 1**: Corresponding anatomical areas, volume, statistical significance and coordinates of clusters within the frontal executive network (FEN), frontoparietal network (FPN) and default mode network (DMN).

| **Area** | **Brodmann Area** | **Volume (cc)** | **Max Value (x, y, z)** | | **MNI (x, y, z)** |
| --- | --- | --- | --- | --- | --- |
| *FEN-positive:* |  |  |  | |  |
| * | * | 0.2/0.6 | 3.0 (-51, 13, -2)/3.8 (2, 24, 54) | | (-52, 14, -2)/(2, 22, 60) |
| Superior Frontal Gyrus | 6, 8, 10 | 7.2/6.9 | 8.1 (-4, 1, 64)/8.6 (2, 5, 62) | | (-4, -2, 70)/(2, 2, 68) |
| Medial Frontal Gyrus | 6, 8, 32 | 3.8/3.4 | 7.2 (-4, 1, 61)/7.4 (4, 1, 61) | | (-4, -2, 66)/(4, -2, 66) |
| Middle Frontal Gyrus | 6, 8, 9 | 4.7/2.3 | 5.2 (-14, 13, 62)/5.7 (18, 9, 62) | | (-14, 10, 68)/(18, 6, 68) |
| Cingulate Gyrus | 24, 32 | 3.0/3.5 | 4.5 (0, 13, 32)/4.9 (2, 17, 34) | | (0, 12, 36)/(2, 16, 38) |
| Inferior Frontal Gyrus | 9, 47 | 0.4/1.0 | 2.8 (-50, 17, -6)/4.6 (53, 15, -6) | | (-50, 18, -6)/(54, 16, -6) |
| Superior Temporal Gyrus | 22, 38 | 1.1/1.2 | 3.9 (-51, 13, -6)/4.2 (57, 13, -4) | | (-52, 14, -6)/(58, 14, -4) |
| Precentral Gyrus | 4, 6 | 1.4/0.0 | 3.9 (-46, -1, 52)/-999.0 (0, 0, 0) | | (-46, -4, 56)/(0, 0, 0) |
| Sub-Gyral | 6 | 0.8/0.3 | 3.8 (-26, -1, 57)/3.3 (18, 5, 57) | | (-26, -4, 62)/(18, 2, 62) |
| Anterior Cingulate | 24, 32 | 0.4/0.4 | 2.8 (-2, 26, 23)/2.5 (4, 23, 23) | | (-2, 26, 26)/(4, 22, 26) |
| Insula | 13 | 0.0/0.2 | -999.0 (0, 0, 0)/2.2 (42, 15, -2) | | (0, 0, 0)/(42, 16, -2) |
| Declive | * | 0.1/0.0 | 2.1 (-38, -59, -21)/-999.0 (0, 0, 0) | | (-38, -60, -28)/(0, 0, 0) |
| Culmen | * | 0.1/0.1 | 2.0 (-36, -56, -22)/2.0 (36, -56, -24) | | (-36, -56, -30)/(36, -56, -32) |
| *FEN-negative:* |  |  |  | |  |
| Precentral Gyrus | 4, 6 | 0.0/2.6 | -999.0 (0, 0, 0)/3.2 (36, -22, 64) | | (0, 0, 0)/(36, -26, 68) |
| * | * | 0.0/0.0 | -999.0 (0, 0, 0)/-999.0 (0, 0, 0) | | (0, 0, 0)/(0, 0, 0) |
| Inferior Frontal Gyrus | 10, 45, 46, 47 | 2.8/0.0 | 3.0 (-50, 37, 0)/-999.0 (0, 0, 0) | | (-50, 38, 2)/(0, 0, 0) |
| Postcentral Gyrus | 1, 2, 3, 5, 40 | 0.0/2.9 | -999.0 (0, 0, 0)/2.9 (34, -30, 64) | | (0, 0, 0)/(34, -34, 68) |
| Middle Frontal Gyrus | 10, 11, 47 | 1.3/0.0 | 2.9 (-50, 37, -4)/-999.0 (0, 0, 0) | | (-50, 38, -2)/(0, 0, 0) |
| Tuber | * | 0.0/0.4 | -999.0 (0, 0, 0)/2.5 (36, -77, -30) | | (0, 0, 0)/(36, -78, -40) |
| Pyramis | * | 0.0/0.1 | -999.0 (0, 0, 0)/2.4 (38, -73, -32) | | (0, 0, 0)/(38, -74, -42) |
| Medial Frontal Gyrus | 6, 8, 9 | 0.3/0.3 | 2.3 (-4, 50, 36)/2.3 (2, -25, 53) | | (-4, 50, 42)/(2, -28, 56) |
| Paracentral Lobule | 5, 6 | 0.1/0.2 | 2.1 (-2, -28, 53)/2.2 (2, -28, 53) | | (-2, -32, 56)/(2, -32, 56) |
| Superior Frontal Gyrus | 8, 9 | 0.5/0.2 | 2.2 (-12, 46, 36)/2.1 (14, 47, 38) | | (-12, 46, 42)/(14, 46, 44) |
| Uvula | * | 0.0/0.1 | -999.0 (0, 0, 0)/2.0 (34, -79, -26) | | (0, 0, 0)/(34, -80, -36) |
| *rFPN-positive* |  |  |  | |  |
| * | * | 0.4/0.1 | 3.2 (-4, -80, -16)/2.7 (6, -67, 57) | | (-4, -82, -24)/(6, -72, 58) |
| Inferior Parietal Lobule | 7, 39, 40 | 1.4/11.2 | 3.3 (-42, -50, 56)/9.3 (44, -50, 54) | | (-42, -54, 58)/(44, -54, 56) |
| Superior Parietal Lobule | 7, 40 | 0.9/4.4 | 3.0 (-30, -63, 57)/9.1 (36, -63, 51) | | (-30, -68, 58)/(36, -68, 52) |
| Precuneus | 7, 19, 39 | 0.0/2.8 | -999.0 (0, 0, 0)/6.1 (26, -69, 51) | | (0, 0, 0)/(26, -74, 52) |
| Middle Frontal Gyrus | 6, 8, 9, 10, 46, 47 | 0.0/21.3 | -999.0 (0, 0, 0)/5.9 (44, 18, 47) | | (0, 0, 0)/(44, 16, 52) |
| Postcentral Gyrus | 1, 2, 5, 40 | 0.0/1.9 | -999.0 (0, 0, 0)/5.6 (53, -34, 51) | | (0, 0, 0)/(54, -38, 54) |
| Superior Frontal Gyrus | 6, 8, 9, 10 | 0.0/4.0 | -999.0 (0, 0, 0)/5.4 (40, 18, 49) | | (0, 0, 0)/(40, 16, 54) |
| Angular Gyrus | 39 | 0.0/1.6 | -999.0 (0, 0, 0)/4.5 (53, -56, 36) | | (0, 0, 0)/(54, -60, 36) |
| Inferior Frontal Gyrus | 9, 10, 45, 46 | 0.0/3.5 | -999.0 (0, 0, 0)/4.4 (53, 15, 29) | | (0, 0, 0)/(54, 14, 32) |
| Supramarginal Gyrus | 40 | 0.0/3.1 | -999.0 (0, 0, 0)/4.3 (55, -49, 37) | | (0, 0, 0)/(56, -52, 38) |
| Declive | * | 1.6/0.0 | 4.2 (-8, -81, -20)/-999.0 (0, 0, 0) | | (-8, -82, -28)/(0, 0, 0) |
| Pyramis | * | 1.3/0.0 | 3.8 (-42, -72, -33)/-999.0 (0, 0, 0) | | (-42, -72, -44)/(0, 0, 0) |
| Uvula | * | 0.4/0.0 | 3.3 (-12, -81, -23)/-999.0 (0, 0, 0) | | (-12, -82, -32)/(0, 0, 0) |
| Precentral Gyrus | 9 | 0.0/0.4 | -999.0 (0, 0, 0)/3.3 (44, 19, 36) | | (0, 0, 0)/(44, 18, 40) |
| Medial Frontal Gyrus | 6, 8, 9 | 0.0/1.2 | -999.0 (0, 0, 0)/3.2 (4, 33, 35) | | (0, 0, 0)/(4, 32, 40) |
| Sub-Gyral | * | 0.0/0.6 | -999.0 (0, 0, 0)/2.9 (34, -56, 42) | | (0, 0, 0)/(34, -60, 42) |
| Tuber | * | 0.7/0.0 | 2.9 (-42, -71, -30)/-999.0 (0, 0, 0) | | (-42, -72, -40)/(0, 0, 0) |
| Cingulate Gyrus | 32 | 0.0/0.3 | -999.0 (0, 0, 0)/2.8 (4, 23, 41) | | (0, 0, 0)/(4, 22, 46) |
| Superior Temporal Gyrus | * | 0.0/0.1 | -999.0 (0, 0, 0)/2.7 (53, -57, 29) | | (0, 0, 0)/(54, -60, 28) |
| Inferior Semi-Lunar Lobule | * | 0.1/0.0 | 2.4 (-44, -64, -35)/-999.0 (0, 0, 0) | | (-44, -64, -46)/(0, 0, 0) |
| Lingual Gyrus | 18 | 0.1/0.0 | 2.3 (-12, -84, -14)/-999.0 (0, 0, 0) | | (-12, -86, -22)/(0, 0, 0) |
| Middle Temporal Gyrus | * | 0.0/0.1 | -999.0 (0, 0, 0)/2.3 (51, -61, 29) | | (0, 0, 0)/(52, -64, 28) |
| *rFPN-negative* |  |  |  | |  |
| Precuneus | 7 | 1.2/0.3 | | 3.7 (-4, -55, 60)/2.4 (2, -52, 56) | (-4, -60, 62)/(2, -56, 58) |
| * | * | 0.0/0.0 | | -999.0 (0, 0, 0)/-999.0 (0, 0, 0) | (0, 0, 0)/(0, 0, 0) |
| Postcentral Gyrus | 7 | 0.3/0.0 | | 3.0 (-4, -49, 67)/-999.0 (0, 0, 0) | (-4, -54, 70)/(0, 0, 0) |
| Superior Frontal Gyrus | 6, 8 | 1.1/0.0 | | 2.5 (-10, 22, 60)/-999.0 (0, 0, 0) | (-10, 20, 66)/(0, 0, 0) |
| Declive | * | 0.0/0.3 | | -999.0 (0, 0, 0)/2.3 (34, -82, -18) | (0, 0, 0)/(34, -84, -26) |
| Uvula | * | 0.0/0.1 | | -999.0 (0, 0, 0)/2.1 (32, -83, -26) | (0, 0, 0)/(32, -84, -36) |
| Paracentral Lobule | 5 | 0.1/0.0 | | 2.0 (-2, -45, 61)/-999.0 (0, 0, 0) | (-2, -50, 64)/(0, 0, 0) |
| Tuber | * | 0.0/0.1 | | -999.0 (0, 0, 0)/2.0 (28, -85, -28) | (0, 0, 0)/(28, -86, -38) |
| *lFPN-positive* |  |  |  | |  |
| Precuneus | 7, 19, 39 | 7.1/0.2 | 7.8 (-28, -69, 50)/2.0 (38, -68, 42) | | (-28, -74, 50)/(38, -72, 42) |
| Superior Parietal Lobule | 7, 40 | 4.4/0.7 | 7.6 (-30, -65, 51)/2.6 (34, -66, 47) | | (-30, -70, 52)/(34, -70, 48) |
| * | * | 0.1/0.1 | 3.3 (-26, 15, 58)/2.3 (6, -79, -16) | | (-26, 12, 64)/(6, -80, -24) |
| Middle Frontal Gyrus | 6, 8, 9, 10, 11, 46, 47 | 20.0/0.0 | 6.5 (-51, 15, 31)/-999.0 (0, 0, 0) | | (-52, 14, 34)/(0, 0, 0) |
| Inferior Parietal Lobule | 39, 40 | 10.8/0.1 | 6.2 (-42, -52, 52)/2.1 (40, -62, 47) | | (-42, -56, 54)/(40, -66, 48) |
| Inferior Frontal Gyrus | 9, 10, 44, 45, 46, 47 | 9.6/0.0 | 5.9 (-50, 11, 31)/-999.0 (0, 0, 0) | | (-50, 10, 34)/(0, 0, 0) |
| Precentral Gyrus | 6, 9, 44 | 0.9/0.0 | 4.0 (-46, 21, 34)/-999.0 (0, 0, 0) | | (-46, 20, 38)/(0, 0, 0) |
| Sub-Gyral | * | 2.6/0.0 | 4.0 (-44, 24, 21)/-999.0 (0, 0, 0) | | (-44, 24, 24)/(0, 0, 0) |
| Angular Gyrus | 39 | 1.7/0.0 | 3.6 (-44, -66, 36)/-999.0 (0, 0, 0) | | (-44, -70, 36)/(0, 0, 0) |
| Superior Frontal Gyrus | 6, 8, 9, 10 | 3.4/0.0 | 3.6 (-28, 14, 51)/-999.0 (0, 0, 0) | | (-28, 12, 56)/(0, 0, 0) |
| Declive | * | 0.0/1.3 | -999.0 (0, 0, 0)/3.3 (10, -79, -21) | | (0, 0, 0)/(10, -80, -30) |
| Postcentral Gyrus | 2, 40 | 0.5/0.0 | 3.1 (-50, -33, 48)/-999.0 (0, 0, 0) | | (-50, -36, 50)/(0, 0, 0) |
| Pyramis | * | 0.0/1.2 | -999.0 (0, 0, 0)/3.1 (42, -72, -33) | | (0, 0, 0)/(42, -72, -44) |
| Supramarginal Gyrus | 40 | 1.2/0.0 | 3.0 (-51, -49, 37)/-999.0 (0, 0, 0) | | (-52, -52, 38)/(0, 0, 0) |
| Medial Frontal Gyrus | 6, 8, 9 | 0.9/0.0 | 3.0 (-2, 27, 37)/-999.0 (0, 0, 0) | | (-2, 26, 42)/(0, 0, 0) |
| Cingulate Gyrus | 32 | 0.4/0.0 | 2.9 (-2, 23, 39)/-999.0 (0, 0, 0) | | (-2, 22, 44)/(0, 0, 0) |
| Uvula | * | 0.0/0.4 | -999.0 (0, 0, 0)/2.5 (14, -81, -25) | | (0, 0, 0)/(14, -82, -34) |
| Tuber | * | 0.0/0.1 | -999.0 (0, 0, 0)/2.4 (42, -71, -30) | | (0, 0, 0)/(42, -72, -40) |
| *lFPN-negative:* |  |  |  | |  |
| Superior Frontal Gyrus | 6, 8, 9 | 0.0/4.1 | -999.0 (0, 0, 0)/3.3 (8, 11, 66) | | (0, 0, 0)/(8, 8, 72) |
| * | * | 0.0/0.2 | -999.0 (0, 0, 0)/2.5 (4, 51, 42) | | (0, 0, 0)/(4, 50, 48) |
| Precuneus | 7 | 0.0/0.8 | -999.0 (0, 0, 0)/3.0 (6, -55, 62) | | (0, 0, 0)/(6, -60, 64) |
| Declive | * | 0.5/0.0 | 2.7 (-24, -88, -19)/-999.0 (0, 0, 0) | | (-24, -90, -28)/(0, 0, 0) |
| Fusiform Gyrus | * | 0.1/0.0 | 2.5 (-20, -88, -17)/-999.0 (0, 0, 0) | | (-20, -90, -26)/(0, 0, 0) |
| Postcentral Gyrus | 5, 7 | 0.0/0.3 | -999.0 (0, 0, 0)/2.5 (8, -49, 65) | | (0, 0, 0)/(8, -54, 68) |
| Uvula | * | 0.3/0.0 | 2.2 (-28, -85, -23)/-999.0 (0, 0, 0) | | (-28, -86, -32)/(0, 0, 0) |
| Inferior Frontal Gyrus | * | 0.0/0.1 | -999.0 (0, 0, 0)/2.1 (36, 20, -18) | | (0, 0, 0)/(36, 22, -20) |
| Medial Frontal Gyrus | 8 | 0.0/0.1 | -999.0 (0, 0, 0)/2.1 (6, 56, 34) | | (0, 0, 0)/(6, 56, 40) |
| Middle Frontal Gyrus | 6 | 0.0/0.1 | -999.0 (0, 0, 0)/2.0 (20, 13, 62) | | (0, 0, 0)/(20, 10, 68) |
| *DMN-positive* |  |  |  | |  |
| Posterior Cingulate | 23, 29, 30, 31 | 5.5/5.5 | 10.2 (0, -49, 21)/9.3 (4, -50, 17) | | (0, -52, 20)/(4, -52, 16) |
| * | * | 0.0/0.1 | -999.0 (0, 0, 0)/3.0 (4, 60, -10) | | (0, 0, 0)/(4, 62, -8) |
| Cingulate Gyrus | 23, 31 | 3.3/2.7 | 7.3 (0, -51, 28)/6.5 (4, -49, 28) | | (0, -54, 28)/(4, -52, 28) |
| Precuneus | 7, 19, 23, 31, 39 | 7.5/3.9 | 7.3 (-2, -59, 18)/5.6 (4, -51, 32) | | (-2, -62, 16)/(4, -54, 32) |
| Sub-Gyral | * | 1.2/1.2 | 6.5 (-2, -44, 10)/4.9 (12, -57, 18) | | (-2, -46, 8)/(12, -60, 16) |
| Extra-Nuclear | * | 1.2/1.2 | 6.2 (-12, -57, 19)/5.2 (16, -53, 21) | | (-12, -60, 18)/(16, -56, 20) |
| Cuneus | 7 | 0.3/0.1 | 4.4 (0, -64, 33)/3.7 (4, -64, 33) | | (0, -68, 32)/(4, -68, 32) |
| Lingual Gyrus | 18, 19 | 0.3/0.0 | 4.1 (-12, -52, 4)/-999.0 (0, 0, 0) | | (-12, -54, 2)/(0, 0, 0) |
| Middle Temporal Gyrus | 19, 22, 39 | 3.4/2.3 | 4.1 (-50, -61, 23)/4.0 (51, -61, 25) | | (-50, -64, 22)/(52, -64, 24) |
| Parahippocampal Gyrus | 30 | 0.3/0.1 | 4.0 (-10, -48, 2)/4.0 (10, -46, 4) | | (-10, -50, 0)/(10, -48, 2) |
| Superior Temporal Gyrus | 22, 39 | 2.9/3.0 | 3.9 (-51, -59, 20)/3.9 (51, -59, 21) | | (-52, -62, 18)/(52, -62, 20) |
| Medial Frontal Gyrus | 10, 11 | 2.0/1.8 | 3.8 (-4, 52, -13)/3.8 (4, 52, -13) | | (-4, 54, -12)/(4, 54, -12) |
| Angular Gyrus | 39 | 1.8/1.0 | 3.6 (-46, -64, 31)/3.3 (46, -64, 33) | | (-46, -68, 30)/(46, -68, 32) |
| Supramarginal Gyrus | 40 | 0.6/0.6 | 3.2 (-48, -53, 23)/2.8 (51, -53, 25) | | (-48, -56, 22)/(52, -56, 24) |
| Inferior Parietal Lobule | 39, 40 | 0.6/0.0 | 3.0 (-42, -68, 38)/-999.0 (0, 0, 0) | | (-42, -72, 38)/(0, 0, 0) |
| Culmen | * | 0.2/0.1 | 2.5 (-2, -48, 2)/2.9 (6, -47, 2) | | (-2, -50, 0)/(6, -48, 0) |
| Superior Frontal Gyrus | 10 | 0.2/0.0 | 2.9 (-4, 60, -10)/-999.0 (0, 0, 0) | | (-4, 62, -8)/(0, 0, 0) |
| Superior Parietal Lobule | 7 | 0.1/0.0 | 2.3 (-36, -70, 44)/-999.0 (0, 0, 0) | | (-36, -74, 44)/(0, 0, 0) |
| Middle Frontal Gyrus | 8 | 0.1/0.0 | 2.1 (-24, 31, 44)/-999.0 (0, 0, 0) | | (-24, 30, 50)/(0, 0, 0) |
| Anterior Cingulate | * | 0.1/0.1 | 2.1 (-4, 40, -9)/2.0 (4, 40, -9) | | (-4, 42, -8)/(4, 42, -8) |
| *DMN-negative* |  |  |  | |  |
| Precuneus | 7, 19 | 0.2/0.8 | 2.3 (-8, -80, 41)/2.9 (10, -77, 44) | | (-8, -84, 40)/(10, -82, 44) |
| * | * | 0.0/0.1 | -999.0 (0, 0, 0)/2.7 (10, -75, 48) | | (0, 0, 0)/(10, -80, 48) |
| Extra-Nuclear | * | 0.1/0.1 | 2.1 (-4, 21, 1)/2.1 (4, 21, 3) | | (-4, 22, 2)/(4, 22, 4) |
| Superior Temporal Gyrus | 13, 38 | 0.0/0.3 | -999.0 (0, 0, 0)/2.3 (46, 3, -10) | | (0, 0, 0)/(46, 4, -12) |
| Cuneus | * | 0.1/0.1 | 2.1 (-8, -82, 37)/2.0 (12, -84, 36) | | (-8, -86, 36)/(12, -88, 34) |
| Inferior Frontal Gyrus | * | 0.0/0.1 | -999.0 (0, 0, 0)/2.0 (36, 23, -8) | | (0, 0, 0)/(36, 24, -8) |

Note: Spatial maps are thresholded at Z=2.
*Clusters are not corresponding with a Brodmann area.
